# A Systematic Screen Reveals MicroRNA Clusters That Significantly Regulate Four Major Signaling Pathways

**DOI:** 10.1371/journal.pone.0048474

**Published:** 2012-11-08

**Authors:** Lindsey E. Becker, Zhongxin Lu, Weiqun Chen, Wei Xiong, Maiying Kong, Yong Li

**Affiliations:** 1 Department of Biochemistry and Molecular Biology, School of Medicine, University of Louisville, Louisville, Kentucky, United States of America; 2 Department of Medical Laboratory and Central Laboratory, The Central Hospital of Wuhan, Wuhan, Hubei, China; 3 Cancer Research Institute, Central South University, Changsha, Hunan, China; 4 Department of Bioinformatics and Biostatistics, School of Public Health and Information Sciences, University of Louisville, Louisville, Kentucky, United States of America; Northwestern University, United States of America

## Abstract

MicroRNAs (miRNAs) are encoded in the genome as individual miRNA genes or as gene clusters transcribed as polycistronic units. About 50% of all miRNAs are estimated to be co-expressed with neighboring miRNAs. Recent studies have begun to illuminate the importance of the clustering of miRNAs from an evolutionary, as well as a functional standpoint. Many miRNA clusters coordinately regulate multiple members of cellular signaling pathways or protein interaction networks. This cooperative method of targeting could produce effects on an overall process that are much more dramatic than the smaller effects often associated with regulation by an individual miRNA. In this study, we screened 366 human miRNA minigenes to determine their effects on the major signaling pathways culminating in AP-1, NF-κB, c-Myc, or p53 transcriptional activity. By stratifying these data into miRNA clusters, this systematic screen provides experimental evidence for the combined effects of clustered miRNAs on these signaling pathways. We also verify p53 as a direct target of miR-200a. This study is the first to provide a panoramic view of miRNA clusters' effects on cellular pathways.

## Introduction

MicroRNAs (miRNAs) are small RNA molecules 20–25 nucleotides in length. Through complementary base paring, miRNAs bind the 3′ UTR of target mRNAs to post-transcriptionally down-regulate gene expression. Originally discovered in *C. elegans*, the first miRNA was found to be a key regulator of development [1], [2]; however, subsequent studies have revealed a myriad of roles for miRNAs in virtually all biological processes. Studies highlighting the biological function of miRNAs have emerged alongside studies that reveal the detrimental effects of miRNA deregulation [3]. Many miRNAs, when lost or over-expressed, become crucial players in the oncogenic process [4], [5]. miRNAs may target a wide variety of genes, including those most closely associated with the processes of cancer development, particularly the hallmarks of cancer [6], [7]. By inhibiting expression of tumor suppressors, miRNAs may function as oncogenes. Conversely, miRNAs can also exhibit tumor suppressive properties by repressing oncogenes.

miRNAs are transcribed and processed from intronic or intergenic regions, and may be transcribed as individual miRNA or as polycistronic transcripts (clusters) [2], [8]. Primary miRNA transcripts (pri-miRNA) are processed into imperfect stem-loop structures called pre-miRNAs by Drosha in the nucleus and then exported into the cytoplasm by Exportin V. These pre-miRNAs are cleaved by Dicer to form mature miRNAs, which are then incorporated into the RNA-induced silencing complex (RISC). Imperfect complementary base-paring between the miRNA and mRNA directs the RISC to the 3′ UTR of target mRNA. This targeting leads to down-regulation of translation of the mRNA, and is often accompanied by a decrease in mRNA levels [2].

Nearly half of all miRNA genes are within 50 kilobases of another miRNA gene [9]. These clusters range from 2 miRNAs, for example miR-200c and miR-141, to as many as 46 miRNAs, as seen in the largest miRNA cluster in primates, Chromosome 19 miRNA Cluster (C19MC) [9]–[11]. miRNAs within clusters frequently contain high sequence homology, particularly within the seed sequence, resulting in identical targets [12], [13]. Recent evidence, however, points to clustered miRNAs that target different genes within a specific pathway or protein complex [14], [15]. miRNAs are also predicted to target downstream effectors of cellular signaling pathways such as second messengers and transcription factors (TFs) more frequently than upstream ligands and receptors or housekeeping and structural genes [16]. TFs are key players in cell signaling pathways. By responding to a plethora of extra- or intra-cellular stimuli and regulating transcription of the many genes necessary for a cellular response, TFs act as crucial cell signaling hubs. Deregulation of major TFs is often a key event in oncogenesis [17]. Such TFs include AP-1, NF-κB, c-Myc, and p53 [18]–[21]. Many individual miRNAs target these pathways [22]–[25], but little data exists regarding the full effect of miRNA clusters. While it is clear that miRNA clusters are frequently predicted to target specific cell signaling pathways, no experimental evidence based on systematic screening has been provided. In this study, we intend to address these deficiencies by analyzing the role of 366 human miRNAs as clusters in these four major signaling pathways using an existing genetic library [26].

## Experimental Procedures

### miRNA Screen

The method involves a published lentiviral-based miRNA genetic library that contains a large number of human miRNA minigenes [26]. To screen miRNAs that specifically target TFs of interest, we utilized luciferase constructs plus the miRNA library. For instance, pTRF-p53-Luc (Systems Biosciences) contains a firefly *luciferase* gene (*luc*) under the control of a minimal CMV promoter. This promoter is only activated when p53 binds to the p53-specific transcription response elements (TREs), eight tandem repeats of ACATGTCCCAACATGTTGTCG. Similarly, TRE constructs for the other TFs are as follows: pTRF-NF-κB-Luc: four repeats of GGGGACTTTCC; and pTRF-AP1-Luc: four repeats of TCCGGTGACTCAGTCAAGCG. c-Myc activity was measured using an E2F2-Luc reporter vector consisting of the E2F2 promotor with four distinct E-boxes, CACGTG [27]. The parental vector, pSIF [26], substituted for the miRNA construct, serves as a normalization control for miRNA expression. *Rluc* from pRL-TK (Promega) is used to normalize transfection efficiency and total protein synthesis.

### Cell Culture Experiments

293T and H1299 cells (American Type Culture Collection, Manassas, VA) were cultured in DMEM media supplemented with 10% FBS and antibiotics at 37°C with 5% CO_2_. Lipofectamine LTX (Invitrogen) was used for all transfections according to manufacturer's instructions. Luciferase assays were conducted using the Dual-Glo® Luciferase Assay System (Promega) 48 hours post-transfection in 96-well plates. Relative Luciferse Units (RLU) were normalized to Renilla luciferase expression. The parental vector pSIF was used to normalize plate-to-plate variation. Apoptosis was measured using an ApoTarget™ Annexin-V FITC Apoptosis Kit (Invitrogen, Carlsbad, CA) as described previously [26]. Briefly, transfected cells were washed twice with PBS, resuspended in Annexin-V binding buffer, and then incubated in Annexin-V FITC and Propidium Iodide Buffer in the dark for 15 minutes at room temperature. Stained cells were then analyzed on an LSR II flow cytometer (BD Biosciences) using FL1 (FITC) and FL3 (PI) lines. Cell cycle was analyzed as described [28].

### Western Blot

Total protein was isolated from cells in 6-well plates using M-PER mammalian protein extraction reagent (PIERCE, Rockford, IL). Protein concentration was measured using a BCA kit (PIERCE, Rockford, IL). 30–50 µg of protein were separated on 12% to 15% Bis-Tris polyacrylamide gels (Bio-Rad, Hercules, CA) and then transferred to PVDF membranes (Bio-Rad). Protein membranes were incubated in blocking buffer (1× Tris-buffered saline, pH 7.5, 5% nonfat dried milk, 0.05% Tween 20) for 2 hours at room temperature, followed by anti-p53 antibody (Santa Cruz Biotechnology, Inc., Santa Cruz, CA), or anti-β-actin antibody (Sigma-Aldrich) overnight at 4°C. The membranes were washed with 1× Tris-buffered saline containing 0.05% Tween 20, incubated with horseradish peroxidase-linked goat anti-mouse Ig (Santa Cruz) or goat anti-Rabbit Ig (Cell Signaling) for 1 hour at room temperature, washed, and visualized with the SuperSignal West Dura/Femto Chemiluminescent Substrate kit (PIERCE).

### Statistical Analysis

Boxplots of the observations for all clusters were plotted to show what the observations look like for each end point variable (AP-1, NF-κB, c-Myc, or p53). For each end point, Residual plots indicated that the observations with log-transformation are more likely to be normally distributed and have equal variances among different clusters. For each variable, one way analysis of variance (ANOVA) was applied to examine whether the observations at log-scale from different clusters are significantly different from the overall means at log scale. Residual plots indicated that the log-transformed responses are more likely to be normally distributed and have equal variances among different clusters. The Fisher's least significant difference tests were applied to examine which clusters are significantly different from the overall least square mean [29]. Based on the analytic results, we painted the boxplots red for the clusters with significantly high readings (observations), and green for the clusters with significantly low readings. The clusters with a pink diamond are significantly different from the overall mean (Figure 2, 3, 4, 5, 6).

## Results

### miRNA Library Screening

**Figure 1:**
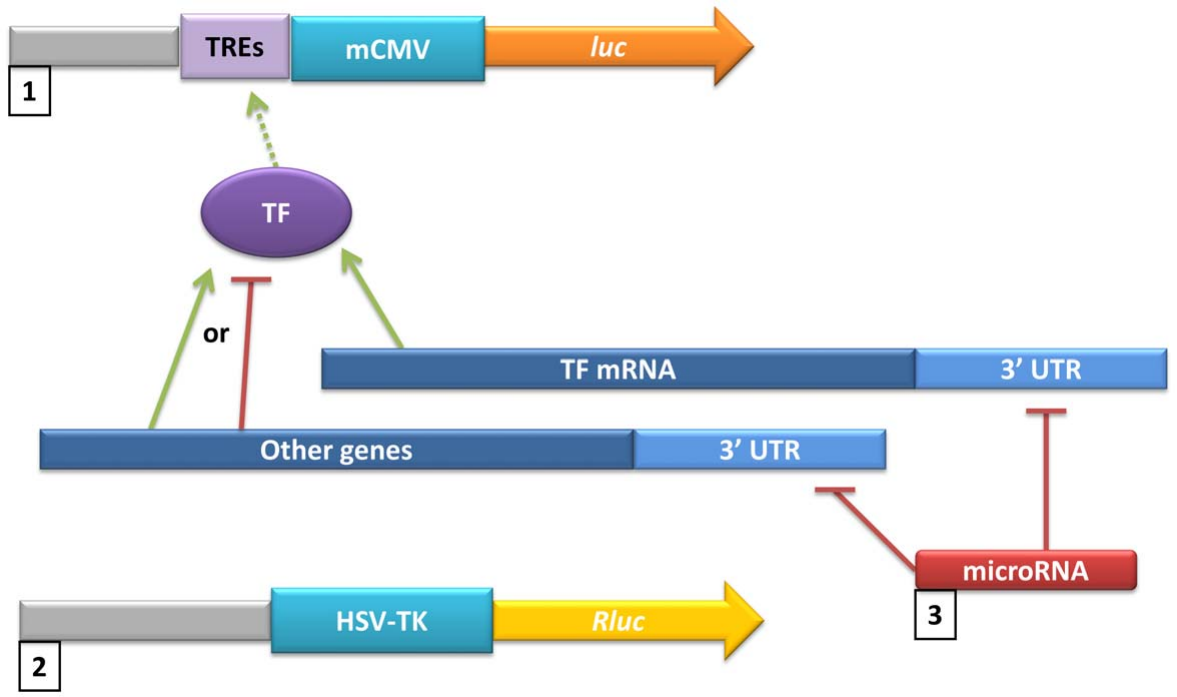
Schematic of luciferase-based microRNA screen. 293T cells were co-transfected with: 1) a vector containing a luciferase gene under control of regulatory elements recognized by AP-1, NF-κB, or p53 (in c-Myc screen, the E2F2-luc construct was used), 2) a member of our microRNA library, and 3) a Renilla luciferase vector for normalization of luciferase values. Following transfection, cells were analyzed by luciferase assay to measure the effects of miRNA regulation of TF-driven luciferase expression. TREs: Transcription response elements, mCMV: minimal CMV promoter, TF: Transcription factor, luc: luciferase, Rluc: Renilla luciferase, UTR: Untranslated region.

We used an established TF luciferase-based screen to determine miRNAs affecting pathways that regulate TF activity (Figure 1). A plasmid containing a firefly luciferase gene under the control of a minimal CMV promoter was transfected into 293T cells along with a second plasmid containing a member of our miRNA library [26]. Transcription response elements (TRE) corresponding to each TF were placed upstream of the promoter. A third plasmid containing a Renilla luciferase gene driven by the HSV-TK promoter served as a normalization control. Luciferase gene expression was measured with a luminometer to determine which miRNA expression resulted in inhibition or promotion of TF activity. Luciferase expression was normalized to Renilla luciferase to yield Relative Luminescence Units (RLU) for each miRNA before being normalized to the parental vector (Table S1). This approach has been used to identify individual miRNAs in the p53, NF-κB, and c-MYC pathways [26], [28], [30]. To analyze the impact of miRNA clusters in reporter activities, mean RLU values for each cluster were calculated and normalized to the mean values of all miRNAs. This allowed us to determine statistical significance of miRNA regulation of specific TFs when miRNA data were stratified into clusters. For each TF, clusters with values significantly lower than the overall cluster mean were identified as down-regulators of the specified TFs. Clusters with values significantly higher than the cluster mean were deemed up-regulators of the specified TF.

### AP-1

**Figure 2:**
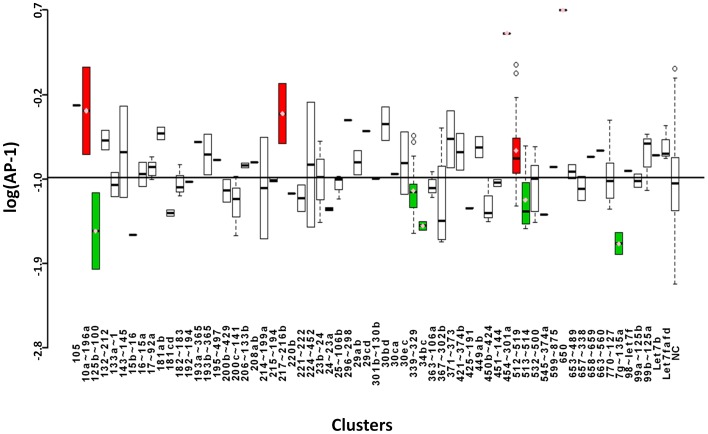
Boxplots showing logarithmic values of luciferase expression for microRNAs grouped according to cluster. Clusters that yielded values significantly different from the overall mean are marked with a pink diamond and annotated in Table 1. MicroRNA clusters that caused significant up-regulation of AP-1-driven luciferase gene expression are highlighted in red. MicroRNA clusters that down-regulated this expression are marked in green.

Activating protein 1 (AP-1) is a dimeric TF consisting of Jun, Fos, or Activating TF (ATF). Combinations of these subunits allow for hetero- and homo-dimerization, resulting in differing DNA recognition and functions of AP-1. The TRE used in this screen is predominantly recognized by the cJun-cFos as well as cJun homodimers to a lesser extent [31], [32]. AP-1 is activated in response to many signals such as stress, bacterial and viral infections, cytokines, growth factors, and oncogenic stimuli. Post-translational regulation occurs through interactions with other TFs, proteolytic turnover, and phosphorylation [31], [33]. Data from the miRNA screen point to five miRNA clusters that yield an overall negative effect on AP-1 directed transcription (Figure 2 and Table 1). These clusters may target genes that are upstream of the pathway directly regulating AP-1 turnover, or genes within signaling cascades that lead to AP-1 activation. Five clusters were found to have an activating effect on AP-1 transcriptional activity. One such noteworthy cluster is 10a∼196a. Studies have established a pro-proliferative role for this cluster in multiple cancers including pancreatic cancer and acute myeloid leukemia [34]–[36]. This role is consistent with our finding that it positively regulates activation of a TF known for its role in promoting proliferation, particularly in the context of cancer [31].

**Table 1:** Top microRNA clusters that significantly modulate reporter expression.

Signaling	Inhibiting Clusters	Difference from the mean	p-value	Activating Clusters	Difference from the mean	p-value
**AP-1**	Let7g∼135a	−0.781	5.14E-03	512∼519a	0.184	1.78E-02
	125b∼100	−0.652	1.92E-02	217∼216b	0.567	4.14E-02
	34bc	−0.595	3.26E-02	10a∼196a	0.597	3.20E-02
	513∼514	−0.329	3.05E-02	454∼301a	1.400	3.86E-04
	339∼329	−0.238	3.22E-03	650	1.636	3.57E-05
**NF-κB**	215∼194	−1.174	1.90E-04	99b∼125a	0.823	2.81E-03
	30bd	−0.874	9.13E-03	181cd	0.840	1.22E-02
	125b∼100	−0.756	2.40E-02	192∼194	1.167	1.33E-02
	206∼133b	−0.744	2.62E-02	650*	1.262	7.49E-03
	217∼216b	−0.681	4.17E-03	454∼301a	1.877	7.89E-05
	513∼514	−0.413	2.39E-02			
**c-Myc**	195∼497	−0.662	3.15E-02	17∼92a	0.306	1.74E-02
	193b∼365	−0.541	1.35E-02	23b∼24	0.368	4.00E-02
	512∼519a	−0.526	8.16E-17	16∼15a	0.432	4.80E-02
	132∼212	−0.526	1.63E-02	200c∼141	0.590	1.08E-03
				200b∼429	0.312	5.25E-03
**p53**	200b∼429	−0.833	6.44E-04	532∼500	0.295	2.61E-02
	30ec	−0.692	4.46E-03	512∼519a	0.444	1.78E-10
	425∼191	−0.616	1.12E-02	99a∼125b	0.523	3.12E-02
	653∼489	−0.568	1.93E-02	296∼298	0.712	3.71E-02
	25∼106b	−0.535	7.25E-03	371∼373	0.748	2.14E-03
				454∼301a	0.766	2.49E-02
				650	0.878	1.03E-02

* miR-650 was included even as it is not in a cluster.

### NF-κB

**Figure 3:**
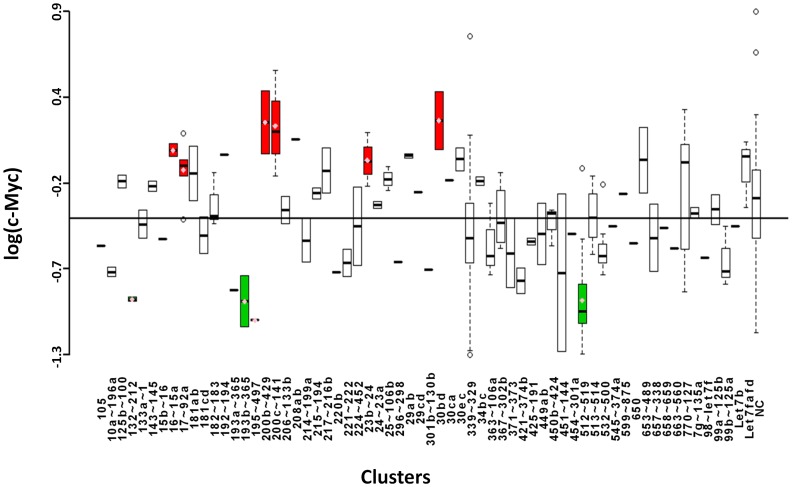
Boxplots showing logarithmic values of NF-κB-induced luciferase expression for microRNAs grouped according to cluster. Annotated as in Figure 2.

NF-κB is a TF that consists of Rel protein dimers that bind κB sites in the promoters of target genes to regulate transcription. The Rel family of proteins consists of five members: p100 and p105 which are proteolytically processed into p50 and p52, respectively, and RelA, RelB, and c-Rel, which do not require proteolytic processing. The TRE in this screen is specifically recognized by the heterodimer made up of p50 and RelA, which is the most abundant form of NF-κB in most cells. This heterodimer is held inactive in the cytoplasm by inhibitors of κB (IκB) [23]. The classical pathway of NF-κB activation is triggered by exposure to bacterial or viral infections and pro-inflammatory cytokines such as TNF-α. These signals go through the Toll-like receptor (TLR) to activate IκB kinases (IKK) which phosphorylate IκB, targeting it for ubiquitin-mediated degradation. NF-κB is released and translocates to the nucleus to promote transcription [23], [37]. One of the major functions of NF-κB is inhibition of apoptosis, though its role in cancer development and progression is cell-type dependent. Suppression of NF-κB activation abrogates transcription of critical anti-apoptotic genes such as c-FIIP, cIAP1, cIAP2, and BCL-X_L_ [37]. This screen revealed seven clusters that negatively regulate NF-κB-mediated transcription (Figure 3 and Table 1). Inhibition of NF-κB signaling implies a potential anti-inflammatory role for these clusters. Five clusters were found to up-regulate NF-κB activity. Among these is cluster 454∼301a. miR-301a has recently been implicated as an NF-κB inducer in pancreatic cancer [26]. Cluster 99b∼125a was also found to up-regulate NF-κB activity. A recent study found that miR-125a and miR-125b directly target TNFAIP3, a ubiquitin editing enzyme that negatively regulates NF-κB activity by disrupting the activation of IKK [38].

### c-Myc

**Figure 4:**
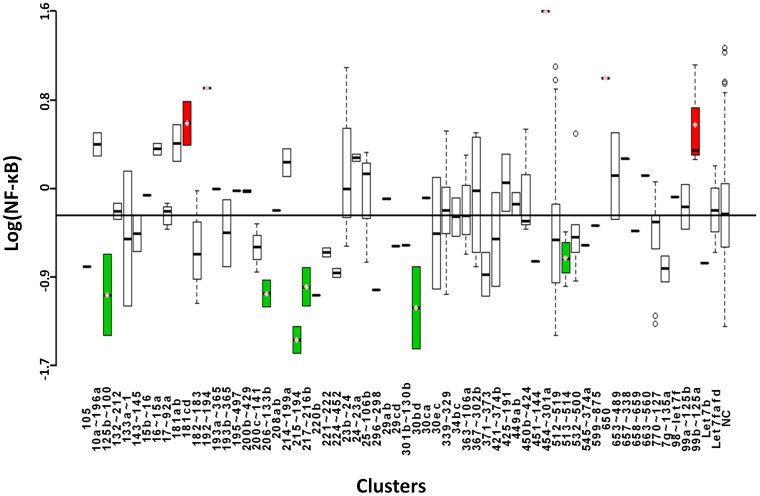
Boxplots showing logarithmic values of c-Myc-induced luciferase expression for microRNAs grouped according to cluster. Annotated as in Figure 2.

c-Myc is a TF that heterodimerizes with Max to bind E-boxes within the promoters of its target genes [39]. It is a multifunctional protein that regulates a wide variety of cellular processes such as cell cycle progression, growth and metabolism, differentiation, and apoptosis [17]. Because of its function in positively regulating processes that contribute to tumorigenesis, Myc is a proto-oncogene. Aberrant expression of Myc is seen in the majority of cancers, resulting from genomic amplification, or lack of negative regulatory pathways [39]. Our screen returned four miRNA clusters that down-regulate Myc-induced transcription (Figure 4 and Table 1). Notably, Cluster 512∼519a negatively regulates Myc-mediated transcriptional activation. Also striking was up-regulation of Myc-mediated transcription by the entire miR-200 family (Clusters 200c∼141 and 200b∼429). In addition, we have confirmed miR-33b as a bona fide c-Myc regulator [30].

### p53

**Figure 5:**
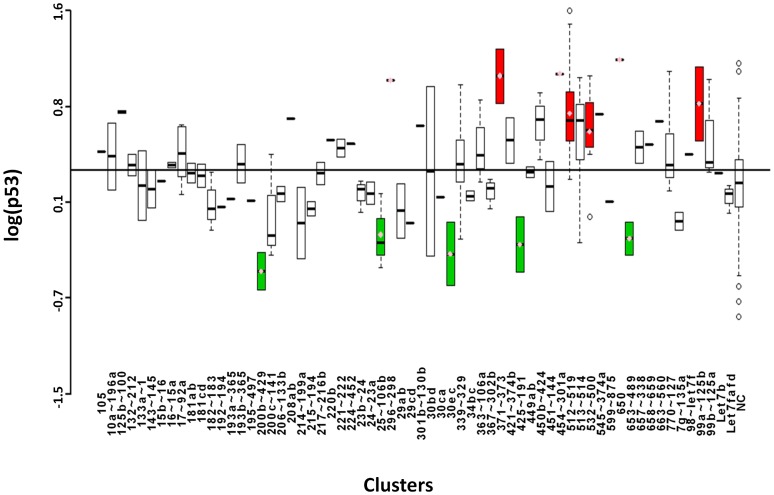
Boxplots showing logarithmic values of p53-induced luciferase expression for microRNAs grouped according to cluster. Annotated as in Figure 2.

p53 has long been known as the guardian of the genome. Its transactivational functions are well studied and include induction of proapoptotic genes like Puma, Noxa, and Bax as well as cell cycle regulatory proteins such as p21 [40]. p53 is maintained at low basal levels in the cell by its inhibitory protein, Mdm2 [41]. Mdm2 inhibits p53 function by acting as an ubiquitin ligase to target p53 for proteasomal degradation as well as by binding and blocking the DNA binding domain of p53, inhibiting its activity as a TF. Upon detection of DNA damage, oncogene hyperactivation, or other cellular stresses, p53 is phosphorylated on its N-terminus, which blocks inhibition by Mdm2 and promotes its binding to p53 response elements. In our screen, we found 7 miRNA clusters that significantly up-regulate p53-mediated luciferase expression (Figure 5 and Table 1). Among these is Cluster512∼519a, also known as C19MC. Comprised of 46 pre-miRNAs, it is the largest miRNA cluster conserved in primates. It is an imprinted gene, and the paternal allele is expressed specifically in the placenta [10], [42]. This tissue specificity is noteworthy in the context of its up-regulation of p53 activity. Enhanced apoptosis and increased p53 expression in the placenta during pregnancy are associated with fetal growth restriction, preeclampsia, intrauterine growth restriction, and HELPP syndrome [43], [44]. Our screen implicates a role for this miRNA cluster within the tightly regulated process of developmental or pathological apoptosis. Among the 5 clusters that down-regulated p53-mediated luciferase expression is 200b∼429, one of two clusters that comprise the miR-200 family (Figure 5 and Table 1). The miR-200 family is largely known as tumor suppressive because of its inhibition of the epithelial-mesenchymal transition (EMT) through direct targeting of Zeb1 and Zeb2 TFs [45], [46]. Our data support an oncogenic role for this miR-200 family and we performed ensuing studies to examine the role of miR-200a in the p53 pathway (see below). Cluster 25∼106b also significantly down-regulated p53 activity, and we noted that miR-25 has been verified to directly target p53 [28].

### miR-200a

The miR-200 family is comprised of two clusters. Cluster 200b∼429 is located on chromosome 1 and contains miR-200a, miR-200b, and miR-429. Cluster 200c∼141 is located on chromosome 12 and contains miR-200c and miR-141. The most thoroughly studied function of the miR-200 family is inhibition of EMT. EMT is characterized by cellular acquisition of mesenchymal morphology and phenotypes and is largely associated with tumor metastasis. In particular, the TFs Zeb1 and Zeb2 are responsible for repressing transcription of E-cadherin and other epithelial markers to promote EMT [47], [48]. The miR-200 family directly targets the 3′ UTRs of Zeb1 and Zeb2 to inhibit their expression, and Zeb1 and Zeb2, on the other hand, bind the promoters of both miR-200 family clusters to reciprocally inhibit their transcription [49]. This miRNA family inhibits proliferation as well as EMT through its targeting of Zeb1 and Zeb2 [50]. Recently, however, new tumor-suppressor targets of the miR-200 family have been discovered, suggesting this miRNA family may have a pro-proliferative function [12], [51], [52]. In addition, a recent study has investigated the miR-200 family's promotion of an epithelial morphology in the context of a mesenchymal-epithelial transition, thus promoting metastatic colonization, and providing further evidence for an oncogenic role for this miRNA family [13].

**Figure 6:**
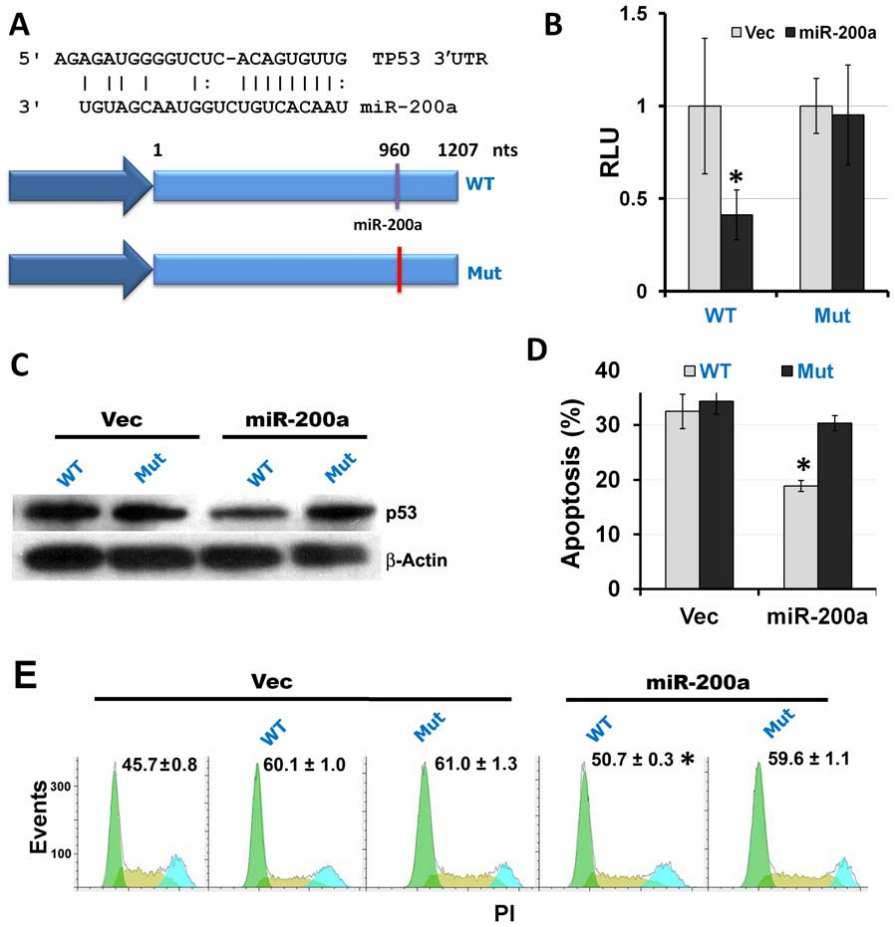
miR-200a directly targets the human p53 gene. A. Schematic representation of miR-200a: p53 3′UTR. Top: seed sequence base paring between miR-200a and the 3′ UTR of p53 mRNA. Bottom: p53 constructs with the wild type miR-200a binding site (WT) or a mutated miR-200 binding site (Mut) in the 3′ UTR. B. A reporter assay to determine whether the p53 3′ UTR is targeted by miR-200a. Y axis denotes relative luminescent units (luc/Rluc) in H1299 cells expressing WT or Mut p53 3′ UTR constructs and miR-200a. C. Western blotting analyses of H1299 cell extracts. H1299 cells were transfected with miR-200a and WT or Mut p53 3′ UTR constructs. D. Apoptosis assay of H1299 cells transfected as in C. E. Cell cycle analysis of H1299 cells transfected as in C. The Y axis denotes events (the number of cells) and the X axis denotes the emitted fluorescent light of the DNA dye (PI), that is, DNA content. The values like “60.1±1.0” indicate the percentages of cells in the G1 phase with standard error of the mean. *P≤0.05 with n = 3.

Our screen revealed a p53-suppressing role for cluster 200b∼429, which contains miR-200a, miR-200b, and miR-429. TargetScan predicts a miR-200a binding site in the 3′ UTR of p53 (Figure 6A). This predicted target is conserved between humans and chimpanzees. To determine direct targeting of p53 by miR-200a, a luciferase assay was performed using constructs with a wild type 3′ p53 UTR (WT) or a 3′ UTR with a mutated miR-200a binding site (Mut) downstream of a luciferase reporter gene. Luciferase assay was performed to measure differential reporter expression resulting from this binding site mutation in p53-null H1299 cells (Figure 6B). Compared to empty vector control, miR-200a caused a significant reduction in WT construct luciferase expression. This reduction of expression was not seen in cells with the mutant 3′ UTR. This suggests that miR-200a directly targets the 3′ UTR of the human p53 gene. Western blot was performed to determine the effects of miR-200a on p53 protein levels. H1299 cells were transfected with miR-200a or its empty vector control, and either p53 coding sequence with a wild type 3′ UTR (WT) or that with a mutated miR-200a binding site in its 3′ UTR (Mut). Compared to the control, miR-200a caused a significant down-regulation of p53 protein levels in cells with a WT 3′ UTR, but not those with a Mut 3′ UTR (Figure 6C). These results show that direct targeting of the p53 3′ UTR by miR-200a down-regulates p53 at the protein level. To determine the functional significance of p53 suppression by miR-200a, we analyzed apoptosis and cell cycle in response to miR-200a over-expression in H1299 cells containing a p53 expression cassette with either WT or Mut 3′ UTR. We found that re-expression of p53 in H1299 cells led to significant cell apoptosis and cell cycle arrest at the G1 phase (G1 arrest) even in the absence of DNA damage (Figure 6D and 6E), in agreement with previous reports [53]–[55]. miR-200a significantly decreased apoptosis in H1299 cells with the WT p53 construct (Figure 6D). Apoptosis was unaffected in cells containing the Mut p53 construct. In addition, G1 arrest was also inhibited by miR-200a compared to the vector control (50.7% *versus* 60.1, *P*≤0.05) only when the exogenous p53 had a WT 3′ UTR (Figure 6E). Taken together, these results provide a new mechanism of oncogenic action for miR-200a. By directly targeting the 3′ UTR of p53, miR-200a down-regulates p53 protein expression, resulting in a significant reduction in apoptosis and G1 arrest.

## Discussion

Most miRNA studies revolve around finding novel targets of single miRNAs, yet half of all miRNAs are co-expressed as clusters [9]. Most of the miRNAs within clusters are likely to be transcribed as a whole unit, so these coexpressed miRNAs shall be investigated together for their biological and pathological function. By stratifying our screen of miRNAs that target TF signaling pathways into miRNA clusters, we were able to collect data that describes the effects of an entire miRNA cluster on a signaling pathway culminating in regulation of a major TF. Several mechanisms exist behind multiple coexpressed miRNAs regulating a wide variety of targets, thus the *modus operandi* of miRNA clusters is not fully understood. Individual miRNAs are predicted to, and have been found to target a wide array of genes and affect multiple cellular functions [6], [56]. Based solely on this, a miRNA cluster could potentially target any and all cell signaling pathways. However, bioinformatics, as well as an increasing number of molecular biology approaches have parsed out a much more ordered pattern of target suppression by miRNA clusters [14], [16], [57], [58]. miRNA clusters are predicted to target interacting members of protein complexes [14], multiple proteins within a single pathway or biological process [16], [57], or multiple clustered miRNAs may simultaneously target and strongly repress a single key regulator of a pathway [59]. In this way, rather than the small scale fine tuning of hundreds of targets [2], a cluster would provide a large combinatorial impact on an entire biological process or pathway. In miRNA clusters comprised of closely related family members, for example both clusters of the miR-200 family or many members of C19MC, similar or same seed sequences provide a clear mechanism for multiple cluster members to target identical sets of genes [13]. This combinatorial system of multiple clustered miRNAs regulating an entire system does not preclude the presence of a single major effector miRNA within a cluster regulating a specific pathway [57]. Xu and Wong propose this mechanism for cluster mmu-miR-183-96-182, which is predicted to control 12 signaling pathways. miR-96 is predicted to target the majority of the genes within these pathways, indicating it as the major effector miRNA of this cluster [57]. Cluster 17∼92a, is a well-studied oncogenic cluster whose most oncogenic member, miR-19, has been experimentally validated as the most active player in the oncogenic process [60]. The 25∼106b cluster, an ortholog of 17∼92, significantly down-regulated p53 reporter activity. We have verified that miR-25 directly targets the p53 gene [28]. It is noted that each miRNA in the 25∼106b cluster is upregulated in multiple myeloma, a cancer with little p53 mutation [61]. miR-25 is the most significantly upregulated miRNA in multiple myeloma, and its expression is inversely correlated with p53 mRNA levels, suggesting that miR-21 upregulation could be responsible for p53 inactivation in cancers without p53 mutation [28]. How other members of the 25∼106b cluster upregulate p53 transactivational activities, however, remains elusive. Similarly, we have verified that miR-301a up-regulates NF-κB by inhibiting Nkrf [26], yet the role of miR-454 (the other member of the 301a∼454 cluster) in the NF-κB pathway needs further investigation.

We experimentally pursued the down-regulation of p53 activity by cluster 200b∼429 and demonstrated the direct targeting of p53 by miR-200a. miR-200a and its orthologs, miR-200b, miR-200c, and miR-141 were first found tumor suppressors as they inhibit EMT through targeting Zeb1 and Zeb2 [45], [47]. Recently, studies have begun investigating the role of miR-200a in the reverse process, mesenchymal-epithelial transition, which enhances the metastatic potential of cancer cells [13]. Down-regulation of p53 and subsequently apoptosis and G1 arrest by miR-200a illuminates a novel function for this miRNA. This, coupled with emerging studies that show evidence for an oncogenic function for miR-200a and its family members [12], [51], [52], provides a strong foundation for the oncogenic potential of miR-200a.

While our screen provides new, preliminary experimental data regarding the effects of miRNA clusters on TF pathways, there are several limitations that must be considered. First, this screen was performed with a single cell line (293T), which does not account for any bias that may arise from tissue or cell-type specific targeting. Second, our screen may return false negatives or positives because other cellular changes may compromise the luciferase reading. For example, miR-34c is a tumor suppressor, identified as such by its direct targeting and repression of c-Myc [60]. However, cluster 34bc was not found in this screen to down-regulate c-Myc activity. Finally, single transient transfections of miRNA-containing plasmids do not replicate endogenous miRNA levels, which may be subject to further regulation when the entire cluster is expressed. This may bias the screen toward miRNAs that are expressed at low endogenous levels. These limitations can be mitigated by further experimental validation using multiple cell lines or performing miRNA inhibition experiments [28], [30].

To summarize, this study provides a panoramic view of miRNA clusters' effects on AP-1, NF-κB, c-Myc, and p53 signaling pathways and will serve as a base for thorough interrogating the contribution of miRNAs in these pathways.

## Supporting Information

Table S1 **Reporter readout for microRNAs in four signaling pathways.**
(DOCX)
