# Supplementary material for: A Systematic Screen Reveals MicroRNA Clusters That Significantly Regulate Four Major Signaling Pathways
Source: PLoS One. 2012 Nov 8;7(11):e48474. doi: 10.1371/journal.pone.0048474 (PMC3493556; doi:10.1371/journal.pone.0048474)
Supplement: Table S1 — Reporter readout for microRNAs in four signaling pathways. (DOCX) [file pone.0048474.s001.docx]

**Supplemental Table S1 Reporter readout for microRNAs in four signaling pathways**

| MicroRNA | Cluster Name | NF-κB | p53 | AP-1 | c-Myc |
| --- | --- | --- | --- | --- | --- |
| hsa-mir-105-1 | 105 | 0.46674 | 1.60084 | 0.75039 | 0.568339 |
| hsa-mir-650 | 650 | 2.70873 | 3.36003 | 2.01405 | 0.576795 |
| hsa-mir-10a | 10a~196a | 1.62405 | 2.02154 | 1.11673 | 0.4664 |
| hsa-mir-196a-1 | 10a~196a | 1.31436 | 1.17041 | 0.45410 | 0.494655 |
| hsa-mir-125b-1 | 125b~100 | 0.24730 | 2.23029 | 0.13712 | 0.827836 |
| hsa-mir-100 | 125b~100 | 0.52398 | 2.17572 | 0.30447 | 0.896151 |
| hsa-mir-132 | 132~212 | 0.72631 | 1.31887 | 0.47427 | 0.398782 |
| hsa-mir-212 | 132~212 | 0.84747 | 1.57032 | 0.58009 | 0.408338 |
| hsa-mir-133a-1 | 133a~1 | 1.13455 | 0.91478 | 0.29141 | 0.714524 |
| hsa-mir-1-1 | 133a~1 | 0.32439 | 1.61360 | 0.37418 | 0.597052 |
| hsa-mir-143 | 143~145 | 0.75054 | 1.38348 | 0.74473 | 0.80987 |
| hsa-mir-145 | 143~145 | 0.53929 | 1.01441 | 0.28942 | 0.862526 |
| hsa-mir-15b | 15b~16 | 0.91231 | 1.26347 | 0.19576 | 0.595387 |
| hsa-mir-16-1 | 16~15a | 1.32357 | 1.46566 | 0.32479 | 1.09662 |
| hsa-mir-15a | 16~15a | 1.47757 | 1.40451 | 0.41820 | 1.009856 |
| hsa-mir-17 | 17~92a | 0.84438 | 1.12753 | 0.41456 | 0.893271 |
| hsa-mir-18a | 17~92a | 0.78599 | 1.51022 | 0.37822 | 0.950848 |
| hsa-mir-19a | 17~92a | 0.78405 | 1.97915 | 0.41581 | 0.955868 |
| hsa-mir-20a | 17~92a | 0.69107 | 1.30684 | 0.34785 | 0.991547 |
| hsa-mir-19b-1 | 17~92a | 0.81671 | 1.95435 | 0.44018 | 1.174425 |
| hsa-mir-18b | 17~92a | 0.66172 | 1.63644 | 0.36449 | 0.672674 |
| hsa-mir-181b-1 | 181ab | 1.74697 | 1.45088 | 0.52928 | 0.759864 |
| hsa-mir-181a-1 | 181ab | 1.24557 | 1.24446 | 0.59912 | 1.078734 |
| hsa-mir-181c | 181cd | 1.44925 | 1.44682 | 0.25446 | 0.684121 |
| hsa-mir-181d | 181cd | 2.17435 | 1.20212 | 0.23847 | 0.541766 |
| hsa-mir-182 | 182~183 | 0.52404 | 1.00875 | 0.29387 | 0.657523 |
| hsa-mir-96 | 182~183 | 0.33419 | 1.35336 | 0.40833 | 0.688957 |
| hsa-mir-183 | 182~183 | 0.95176 | 0.84692 | 0.32259 | 0.911691 |
| hsa-mir-192 | 192~194 | 2.46289 | 1.02305 | 0.34127 | 1.024604 |
| hsa-mir-193a | 193a~365 | 0.96220 | 1.09407 | 0.51340 | 0.427066 |
| hsa-mir-193b | 193b~365 | 0.87433 | 1.69275 | 0.55836 | 0.468104 |
| hsa-mir-365-1 | 193b~365 | 0.46771 | 1.23893 | 0.36652 | 0.337391 |
| hsa-mir-497 | 195~497 | 0.94420 | 1.07670 | 0.42845 | 0.352075 |
| hsa-mir-200a | 200b~409 | 0.95371 | 0.70808 | 0.34853 | 1.029777 |
| hsa-mir-429 | 200b~409 | 0.92940 | 0.52120 | 0.27701 | 1.541511 |
| hsa-mir-200b | 200c~141 | 0.56206 | 0.81121 | 0.35980 | 0.891762 |
| hsa-mir-200c | 200c~141 | 0.44369 | 1.56166 | 0.28545 | 1.186587 |
| hsa-mir-141 | 200c~141 | 0.69899 | 0.69463 | 0.19556 | 1.76595 |
| hsa-mir-206 | 206~133b | 0.41201 | 1.07049 | 0.41516 | 0.655419 |
| hsa-mir-133b | 206~133b | 0.32184 | 1.20463 | 0.39454 | 0.780515 |
| hsa-mir-208 | 208ab | 0.79207 | 2.08890 | 0.41584 | 1.13054 |
| hsa-mir-214 | 214~199a | 1.08661 | 0.67399 | 0.54097 | 0.677515 |
| hsa-mir-199a-1 | 214~199a | 1.39667 | 1.19690 | 0.18870 | 0.511949 |
| hsa-mir-215 | 215~194 | 0.20896 | 1.06540 | 0.35357 | 0.828119 |
| hsa-mir-194-1 | 215~194 | 0.26862 | 0.95373 | 0.34182 | 0.768515 |
| hsa-mir-217 | 217~216b | 0.46427 | 1.46663 | 0.94222 | 0.798145 |
| hsa-mir-216 | 217~216b | 0.32389 | 1.22521 | 0.50759 | 1.071246 |
| hsa-mir-220 | 220b | 0.35878 | 1.75204 | 0.30259 | 0.47934 |
| hsa-mir-221 | 221~222 | 0.55854 | 1.52709 | 0.32863 | 0.467678 |
| hsa-mir-222 | 221~222 | 0.51249 | 1.77200 | 0.25063 | 0.556502 |
| hsa-mir-224 | 224~452 | 0.42545 | 1.69920 | 0.21359 | 0.831109 |
| hsa-mir-452 | 224~452 | 0.46036 | 1.70182 | 0.77901 | 0.501935 |
| hsa-mir-23b | 23b~24 | 0.96360 | 0.97700 | 0.35774 | 0.833963 |
| hsa-mir-27b | 23b~24 | 0.56427 | 1.17800 | 0.51871 | 0.978232 |
| hsa-mir-24-1 | 23b~24 | 2.97664 | 1.26366 | 0.22400 | 1.178903 |
| hsa-mir-27a | 24~23a | 1.24690 | 1.25173 | 0.26063 | 0.723743 |
| hsa-mir-23a | 24~23a | 1.32908 | 1.04220 | 0.25218 | 0.757501 |
| hsa-mir-25 | 25~106b | 1.35206 | 0.62723 | 0.34916 | 0.873251 |
| hsa-mir-93 | 25~106b | 0.48819 | 1.14251 | 0.28503 | 0.812482 |
| hsa-mir-106b | 25~106b | 1.10597 | 0.76326 | 0.36190 | 0.949756 |
| hsa-mir-296 | 296~298 | 0.37763 | 2.84719 | 0.64541 | 0.513718 |
| hsa-mir-29a | 29ab | 0.87313 | 1.23678 | 0.47173 | 1.003497 |
| hsa-mir-29b-1 | 29ab | 0.88788 | 0.79292 | 0.36629 | 1.028263 |
| hsa-mir-29c | 29cb | 0.56654 | 0.89835 | 0.57853 | 0.805264 |
| hsa-mir-130b | 301b~130b | 0.57156 | 1.97275 | 0.35234 | 0.487416 |
| hsa-mir-30b | 30bd | 0.21802 | 2.70063 | 0.74086 | 1.057599 |
| hsa-mir-30d | 30bd | 0.46905 | 0.68673 | 0.52208 | 1.536936 |
| hsa-mir-30a | 30ca | 0.88505 | 1.10511 | 0.37054 | 0.869461 |
| hsa-mir-30e | 30ec | 1.07022 | 0.90370 | 0.57181 | 0.924255 |
| hsa-mir-30c-1 | 30ec | 0.37949 | 0.54124 | 0.29949 | 1.071697 |
| hsa-mir-32 | 339~329 | 0.81332 | 1.13202 | 0.19938 | 0.819565 |
| hsa-mir-379 | 339~329 | 0.66231 | 1.25663 | 0.33193 | 0.743807 |
| hsa-mir-411 | 339~329 | 0.70224 | 1.46540 | 0.41440 | 0.696166 |
| hsa-mir-299 | 339~329 | 0.83207 | 2.74510 | 0.33548 | 0.5737 |
| hsa-mir-380 | 339~329 | 0.69301 | 1.26915 | 0.28486 | 0.487514 |
| hsa-mir-323 | 339~329 | 0.48820 | 2.04213 | 0.33219 | 0.519747 |
| hsa-mir-329-1 | 339~329 | 0.61043 | 1.67702 | 0.29521 | 0.519728 |
| hsa-mir-494 | 339~329 | 0.85976 | 1.52524 | 0.22725 | 1.159568 |
| hsa-mir-495 | 339~329 | 1.11656 | 1.57295 | 0.32709 | 0.59966 |
| hsa-mir-654 | 339~329 | 0.79187 | 1.35322 | 0.32468 | 0.837718 |
| hsa-mir-376b | 339~329 | 0.81441 | 1.08715 | 0.24535 | 0.474651 |
| hsa-mir-376a-1 | 339~329 | 0.77819 | 1.77656 | 0.24694 | 2.19551 |
| hsa-mir-381 | 339~329 | 1.15680 | 0.78878 | 0.41826 | 0.7844 |
| hsa-mir-487b | 339~329 | 0.72476 | 1.29599 | 0.29810 | 0.598586 |
| hsa-mir-539 | 339~329 | 1.65606 | 1.39037 | 0.44674 | 0.667284 |
| hsa-mir-544 | 339~329 | 1.10563 | 1.44142 | 0.25852 | 0.661799 |
| hsa-mir-655 | 339~329 | 0.97403 | 1.22685 | 0.26146 | 0.595484 |
| hsa-mir-487a | 339~329 | 0.82651 | 1.84126 | 0.30039 | 0.509542 |
| hsa-mir-382 | 339~329 | 0.53422 | 1.41360 | 0.23348 | 0.533171 |
| hsa-mir-134 | 339~329 | 0.46999 | 1.31846 | 0.28870 | 0.752708 |
| hsa-mir-485 | 339~329 | 0.97867 | 0.96017 | 0.27631 | 0.735375 |
| hsa-mir-453 | 339~329 | 1.02711 | 1.03311 | 0.33298 | 0.671911 |
| hsa-mir-154 | 339~329 | 0.60334 | 1.16160 | 0.25290 | 0.461895 |
| hsa-mir-496 | 339~329 | 0.71792 | 1.73906 | 0.30668 | 0.447986 |
| hsa-mir-377 | 339~329 | 0.78548 | 1.24058 | 0.32749 | 0.50891 |
| hsa-mir-409 | 339~329 | 0.87787 | 1.73539 | 0.30951 | 0.463042 |
| hsa-mir-412 | 339~329 | 0.45173 | 2.07251 | 0.25317 | 1.062813 |
| hsa-mir-369 | 339~329 | 1.29319 | 2.17715 | 0.34281 | 0.282116 |
| hsa-mir-410 | 339~329 | 1.38441 | 1.80094 | 0.51569 | 0.288901 |
| hsa-mir-656 | 339~329 | 0.61561 | 2.39157 | 0.54978 | 0.544267 |
| hsa-mir-34b | 34bc | 0.89011 | 1.07159 | 0.22634 | 0.84144 |
| hsa-mir-34c | 34bc | 0.62012 | 1.16004 | 0.20683 | 0.88771 |
| hsa-mir-363 | 363~106a | 1.32471 | 1.55869 | 0.37918 | 0.532022 |
| hsa-mir-20b | 363~106a | 0.75273 | 2.42923 | 0.28951 | 0.472996 |
| hsa-mir-106a | 363~106a | 0.52719 | 1.25478 | 0.31916 | 0.749178 |
| hsa-mir-367 | 367~302b | 0.46901 | 1.20474 | 0.19056 | 0.559943 |
| hsa-mir-302a | 367~302b | 0.60847 | 1.17798 | 0.18334 | 0.604228 |
| hsa-mir-302c | 367~302b | 1.48209 | 1.00484 | 0.26990 | 0.914602 |
| hsa-mir-302b | 367~302b | 1.63188 | 1.28150 | 0.59518 | 0.720165 |
| hsa-mir-371 | 371~373 | 0.35622 | 2.36684 | 0.39380 | 0.434677 |
| hsa-mir-373 | 371~373 | 0.53303 | 3.67911 | 0.71295 | 0.677084 |
| hsa-mir-421 | 421~374b | 0.93259 | 1.46008 | 0.38513 | 0.492101 |
| hsa-mir-374 | 421~374b | 0.39044 | 2.10547 | 0.56079 | 0.41817 |
| hsa-mir-425 | 425~191 | 0.78302 | 0.94207 | 0.25868 | 0.597379 |
| hsa-mir-191 | 425~191 | 1.32948 | 0.60419 | 0.26080 | 0.572908 |
| hsa-mir-449 | 449ab | 0.92880 | 1.30125 | 0.54279 | 0.750514 |
| hsa-mir-449b | 449ab | 0.75262 | 1.41365 | 0.43870 | 0.50412 |
| hsa-mir-542 | 450b~424 | 0.71681 | 1.50190 | 0.24591 | 0.71592 |
| hsa-mir-503 | 450b~424 | 0.66476 | 2.06661 | 0.22470 | 0.568779 |
| hsa-mir-424 | 450b~424 | 1.67959 | 2.58328 | 0.35345 | 0.701926 |
| hsa-mir-451 | 451~144 | 0.49242 | 0.98291 | 0.32505 | 0.287646 |
| hsa-mir-144 | 451~144 | 0.48908 | 1.48162 | 0.34933 | 0.795017 |
| hsa-mir-301 | 454~301a | 5.00789 | 3.00583 | 1.58977 | 0.614213 |
| hsa-mir-512-1 | 512~519a | 1.09280 | 1.29274 | 0.26538 | 0.44233 |
| hsa-mir-498 | 512~519a | 2.66347 | 1.65527 | 0.63269 | 0.936791 |
| hsa-mir-520e | 512~519a | 2.44055 | 2.26697 | 1.14680 | 0.384181 |
| hsa-mir-515-1 | 512~519a | 0.65373 | 1.75498 | 0.42420 | 0.53822 |
| hsa-mir-519e | 512~519a | 0.70165 | 1.27503 | 0.42487 | 0.345763 |
| hsa-mir-520f | 512~519a | 1.11991 | 3.47164 | 0.68458 | 0.41987 |
| hsa-mir-519c | 512~519a | 0.81525 | 2.58806 | 1.04974 | 0.328127 |
| hsa-mir-520a | 512~519a | 0.45212 | 1.68162 | 0.35685 | 0.58379 |
| hsa-mir-526b | 512~519a | 0.24612 | 2.61236 | 0.50256 | 0.594783 |
| hsa-mir-519b | 512~519a | 1.09730 | 2.50092 | 0.76462 | 0.415188 |
| hsa-mir-525 | 512~519a | 0.67524 | 1.86533 | 0.53609 | 0.373102 |
| hsa-mir-523 | 512~519a | 0.60620 | 1.65521 | 0.41901 | 0.334244 |
| hsa-mir-518f | 512~519a | 0.41267 | 1.76239 | 0.37279 | 0.346087 |
| hsa-mir-520b | 512~519a | 0.38647 | 1.67984 | 0.40506 | 0.35311 |
| hsa-mir-518b | 512~519a | 0.89497 | 2.48452 | 0.40913 | 0.328226 |
| hsa-mir-526a-1 | 512~519a | 0.58255 | 2.68249 | 0.43508 | 0.484974 |
| hsa-mir-520c | 512~519a | 0.40461 | 4.98266 | 0.51854 | 0.318227 |
| hsa-mir-518c | 512~519a | 0.42266 | 2.30580 | 0.32608 | 0.460017 |
| hsa-mir-524 | 512~519a | 2.09172 | 1.74744 | 0.44453 | 0.36517 |
| hsa-mir-517a | 512~519a | 0.61408 | 2.01984 | 0.35674 | 0.282893 |
| hsa-mir-519d | 512~519a | 0.29336 | 1.79614 | 0.39827 | 0.432873 |
| hsa-mir-520d | 512~519a | 0.71261 | 3.19491 | 0.81796 | 0.412408 |
| hsa-mir-517b | 512~519a | 0.31309 | 1.29748 | 0.34135 | 0.301642 |
| hsa-mir-520g | 512~519a | 0.59367 | 1.71956 | 0.39065 | 0.300054 |
| hsa-mir-518e | 512~519a | 0.29771 | 3.75036 | 0.46856 | 0.369229 |
| hsa-mir-518a-1 | 512~519a | 0.83542 | 2.21906 | 0.48859 | 0.380043 |
| hsa-mir-518d | 512~519a | 0.44696 | 2.05807 | 0.58465 | 0.443834 |
| hsa-mir-517c | 512~519a | 0.31120 | 1.96188 | 0.52645 | 0.333193 |
| hsa-mir-520h | 512~519a | 0.59517 | 2.14724 | 0.35507 | 0.359493 |
| hsa-mir-521-1 | 512~519a | 0.36341 | 1.87886 | 0.26576 | 0.46283 |
| hsa-mir-522 | 512~519a | 0.60055 | 2.16903 | 0.31001 | 0.444444 |
| hsa-mir-519a-1 | 512~519a | 0.35297 | 4.47454 | 0.53257 | 0.360662 |
| hsa-mir-527 | 512~519a | 3.00097 | 3.22349 | 0.62456 | 0.365952 |
| hsa-mir-513-1 | 513~514 | 0.39141 | 2.43344 | 0.20926 | 0.806952 |
| hsa-mir-506 | 513~514 | 0.47169 | 1.62104 | 0.29994 | 0.614228 |
| hsa-mir-507 | 513~514 | 0.50504 | 0.76827 | 0.23181 | 0.681361 |
| hsa-mir-508 | 513~514 | 0.60057 | 2.91098 | 0.49638 | 0.893243 |
| hsa-mir-509 | 513~514 | 0.41377 | 2.26191 | 0.20903 | 0.584996 |
| hsa-mir-510 | 513~514 | 0.64636 | 1.38881 | 0.24989 | 0.786948 |
| hsa-mir-514-1 | 513~514 | 0.57524 | 2.06203 | 0.37813 | 0.53745 |
| hsa-mir-566 | 532~500 | 0.69290 | 0.94099 | 0.22299 | 0.502929 |
| hsa-mir-532 | 532~500 | 0.41159 | 2.51798 | 0.45397 | 0.539245 |
| hsa-mir-188 | 532~500 | 0.47930 | 1.56182 | 0.23466 | 0.531488 |
| hsa-mir-500 | 532~500 | 0.61373 | 1.90591 | 0.35342 | 0.472937 |
| hsa-mir-362 | 532~500 | 0.68841 | 2.95347 | 0.35996 | 0.516572 |
| hsa-mir-501 | 532~500 | 0.59194 | 1.75711 | 0.26737 | 0.613284 |
| hsa-mir-660 | 532~500 | 1.61485 | 2.23718 | 0.49158 | 0.84555 |
| hsa-mir-545 | 545~374a | 0.56994 | 2.16920 | 0.24297 | 0.645783 |
| hsa-mir-599 | 599~875 | 0.68590 | 1.07094 | 0.39723 | 0.793963 |
| hsa-mir-653 | 653~489 | 1.63094 | 0.90446 | 0.40597 | 1.220565 |
| hsa-mir-489 | 653~489 | 0.72956 | 0.69290 | 0.35078 | 0.800184 |
| hsa-mir-657 | 657~338 | 1.28250 | 1.45235 | 0.35967 | 0.482158 |
| hsa-mir-338 | 657~338 | 1.26834 | 1.88167 | 0.27983 | 0.743149 |
| hsa-mir-658 | 658~659 | 0.65098 | 1.69344 | 0.44055 | 0.637548 |
| hsa-mir-663 | 663~560 | 1.09554 | 2.04387 | 0.47116 | 0.559911 |
| hsa-mir-493 | 770~127 | 0.75135 | 3.06321 | 0.64749 | 1.371609 |
| hsa-mir-337 | 770~127 | 0.86816 | 1.84166 | 0.41230 | 1.095862 |
| hsa-mir-431 | 770~127 | 0.27514 | 3.00042 | 0.51752 | 1.072028 |
| hsa-mir-433 | 770~127 | 0.63539 | 1.30030 | 0.30874 | 0.971729 |
| hsa-mir-127 | 770~127 | 0.74662 | 1.15994 | 0.39566 | 1.169029 |
| hsa-mir-432 | 770~127 | 0.29543 | 1.40369 | 0.25767 | 0.423476 |
| hsa-mir-136 | 770~127 | 0.71063 | 1.24093 | 0.28798 | 0.52029 |
| hsa-mir-370 | 770~127 | 0.55264 | 1.44006 | 0.26243 | 0.557364 |
| hsa-mir-33 | 770~127 | 1.02595 | 1.57986 | 0.34461 | 0.931867 |
| hsa-let-7g | 7g~135a | 0.40571 | 0.84906 | 0.20136 | 0.679732 |
| hsa-mir-135a-1 | 7g~135a | 0.51845 | 0.97688 | 0.16027 | 0.726438 |
| hsa-mir-98 | 98~let7f | 0.89550 | 1.57040 | 0.38143 | 0.526718 |
| hsa-mir-99a | 99a~125b | 1.00672 | 3.17869 | 0.32127 | 0.653871 |
| hsa-let-7c | 99a~125b | 0.66235 | 1.74601 | 0.36864 | 0.789944 |
| hsa-mir-99b | 99b~125a | 1.26505 | 1.35156 | 0.31050 | 0.646226 |
| hsa-let-7e | 99b~125a | 3.06233 | 1.46681 | 0.50600 | 0.445206 |
| hsa-mir-125a | 99b~125a | 1.37257 | 2.87478 | 0.55791 | 0.4822 |
| hsa-let-7b | let7b | 0.48450 | 1.33999 | 0.44981 | 0.646205 |
| hsa-let-7a-1 | let7fafd | 0.53649 | 1.21787 | 0.60865 | 1.110327 |
| hsa-let-7f-1 | let7fafd | 0.78684 | 1.14028 | 0.43594 | 1.011212 |
| hsa-let-7d | let7fafd | 1.19646 | 0.97536 | 0.45527 | 0.726258 |
| hsa-mir-551a |  | 0.33560 | 1.07007 | 0.28690 | 1.066591 |
| hsa-mir-34a |  | 1.29503 | 1.99289 | 0.62911 | 0.731325 |
| hsa-mir-101-1 |  | 0.42579 | 0.62476 | 0.24261 | 0.798405 |
| hsa-mir-186 |  | 0.99017 | 0.89178 | 0.45643 | 0.929527 |
| hsa-mir-137 |  | 2.10647 | 0.94331 | 0.38145 | 0.811604 |
| hsa-mir-553 |  | 0.92050 | 1.13467 | 0.73171 | 0.831505 |
| hsa-mir-197 |  | 0.81142 | 1.29155 | 0.48106 | 0.891277 |
| hsa-mir-554 |  | 0.49872 | 1.02606 | 0.37689 | 0.795485 |
| hsa-mir-555 |  | 0.62287 | 0.94456 | 0.77514 | 0.667768 |
| hsa-mir-9-1 |  | 0.48897 | 0.70354 | 0.82027 | 0.45563 |
| hsa-mir-557 |  | 0.73646 | 0.58542 | 0.37039 | 0.860812 |
| hsa-mir-488 |  | 0.33905 | 1.10734 | 0.57807 | 0.709806 |
| hsa-mir-135b |  | 0.39727 | 0.90225 | 0.38945 | 0.875689 |
| hsa-mir-205 |  | 0.48918 | 1.28574 | 0.65448 | 0.918774 |
| hsa-mir-558 |  | 0.74325 | 1.32714 | 1.00119 | 0.668872 |
| hsa-mir-128a |  | 0.94971 | 0.66128 | 0.52472 | 0.845685 |
| hsa-mir-10b |  | 0.91175 | 1.27453 | 0.63786 | 0.968101 |
| hsa-mir-26b |  | 0.26835 | 1.73995 | 0.58626 | 0.972717 |
| hsa-mir-153-1 |  | 0.42694 | 1.04668 | 0.23564 | 0.606446 |
| hsa-mir-562 |  | 0.38871 | 0.70390 | 0.16695 | 0.679517 |
| hsa-mir-563 |  | 0.82781 | 0.61209 | 0.20133 | 0.847049 |
| hsa-mir-128b |  | 0.91823 | 0.47625 | 0.20880 | 0.727025 |
| hsa-mir-26a-1 |  | 0.89568 | 1.12394 | 0.16791 | 0.719825 |
| hsa-mir-565 |  | 0.70621 | 0.97368 | 0.29904 | 0.626081 |
| hsa-mir-567 |  | 0.77343 | 0.99661 | 0.21835 | 0.631918 |
| hsa-mir-198 |  | 1.81869 | 1.38830 | 0.31734 | 2.568442 |
| hsa-mir-551b |  | 0.48218 | 0.93225 | 0.16600 | 0.584632 |
| hsa-mir-569 |  | 0.51081 | 1.39141 | 0.14114 | 0.687822 |
| hsa-mir-28 |  | 0.65483 | 1.00427 | 0.11944 | 0.703669 |
| hsa-mir-571 |  | 0.56761 | 1.45681 | 0.26075 | 0.644689 |
| hsa-mir-95 |  | 0.61597 | 1.06527 | 0.17591 | 0.645647 |
| hsa-mir-572 |  | 0.49550 | 1.26458 | 0.20809 | 0.597256 |
| hsa-mir-218-1 |  | 0.52866 | 1.41651 | 0.19018 | 0.584798 |
| hsa-mir-573 |  | 0.59097 | 1.36791 | 0.23580 | 0.642435 |
| hsa-mir-575 |  | 1.07292 | 0.76881 | 0.26759 | 0.678161 |
| hsa-mir-576 |  | 1.32749 | 0.98686 | 0.30870 | 0.751268 |
| hsa-mir-577 |  | 0.66033 | 1.00160 | 0.23007 | 0.813086 |
| hsa-mir-579 |  | 0.95317 | 1.10718 | 0.18359 | 0.732586 |
| hsa-mir-581 |  | 0.79554 | 1.10305 | 0.46803 | 0.848894 |
| hsa-mir-583 |  | 0.71812 | 0.89921 | 0.34527 | 0.654996 |
| hsa-mir-584 |  | 0.61457 | 1.04060 | 0.82677 | 0.727115 |
| hsa-mir-378 |  | 0.5459047 | 1.07898 | 0.25199 | 0.972901 |
| hsa-mir-146a |  | 0.48347 | 1.05975 | 0.35045 | 0.939043 |
| hsa-mir-103-1 |  | 0.92148 | 1.15245 | 0.37555 | 0.76969 |
| hsa-mir-340 |  | 0.78566 | 0.78890 | 0.33978 | 0.833976 |
| hsa-mir-219-1 |  | 1.88150 | 1.30795 | 0.52226 | 1.010444 |
| hsa-mir-587 |  | 0.48866 | 1.01667 | 0.62505 | 0.806203 |
| hsa-mir-548b |  | 0.44301 | 1.44893 | 0.54007 | 0.810995 |
| hsa-mir-588 |  | 0.91369 | 1.18017 | 0.46804 | 1.218749 |
| hsa-mir-339 |  | 0.36342 | 1.64161 | 0.32919 | 0.872658 |
| hsa-mir-589 |  | 0.86613 | 1.19215 | 0.30872 | 0.757799 |
| hsa-mir-148a |  | 1.10608 | 1.48658 | 1.10441 | 0.788752 |
| hsa-mir-196b |  | 0.61096 | 1.16760 | 0.53239 | 0.641459 |
| hsa-mir-590 |  | 0.64503 | 0.42224 | 0.22530 | 0.760597 |
| hsa-mir-591 |  | 1.05963 | 0.85013 | 0.31687 | 0.841915 |
| hsa-mir-592 |  | 0.62461 | 0.84419 | 0.25847 | 0.836607 |
| hsa-mir-593 |  | 0.63920 | 0.59240 | 0.27857 | 0.847771 |
| hsa-mir-129-1 |  | 0.50741 | 1.01752 | 0.24677 | 1.051896 |
| hsa-mir-335 |  | 1.04702 | 1.27557 | 0.49408 | 0.936342 |
| hsa-mir-490 |  | 0.95077 | 1.34081 | 0.31416 | 1.178738 |
| hsa-mir-594 |  | 0.65256 | 1.06024 | 0.36509 | 0.855654 |
| hsa-mir-595 |  | 0.67308 | 0.93244 | 0.34922 | 0.699804 |
| hsa-mir-596 |  | 1.41398 | 0.62688 | 0.25167 | 0.866441 |
| hsa-mir-597 |  | 0.46249 | 1.30769 | 0.82190 | 1.973554 |
| hsa-mir-124a-1 |  | 0.66124 | 0.53818 | 0.26819 | 0.427379 |
| hsa-mir-598 |  | 0.66324 | 0.89936 | 0.26537 | 0.765936 |
| hsa-mir-383 |  | 0.52892 | 1.00004 | 0.43930 | 0.775074 |
| hsa-mir-320 |  | 0.72831 | 1.03380 | 0.32880 | 0.948236 |
| hsa-mir-548d-1 |  | 0.77301 | 1.02405 | 0.60925 | 0.909335 |
| hsa-mir-151 |  | 0.41520 | 0.96025 | 0.36269 | 0.631373 |
| hsa-mir-661 |  | 0.45307 | 1.14928 | 0.30810 | 0.837146 |
| hsa-mir-491 |  | 0.47228 | 1.06060 | 0.28881 | 0.92607 |
| hsa-mir-31 |  | 0.49967 | 1.22303 | 0.32805 | 1.187902 |
| hsa-mir-204 |  | 0.40196 | 1.14490 | 0.42352 | 0.817049 |
| hsa-mir-7-1 |  | 0.71520 | 0.72381 | 0.49844 | 0.842641 |
| hsa-mir-455 |  | 0.67092 | 1.05701 | 0.21173 | 0.698667 |
| hsa-mir-147 |  | 1.15964 | 1.19320 | 0.23919 | 0.998777 |
| hsa-mir-600 |  | 0.29308 | 1.69746 | 0.14586 | 1.010761 |
| hsa-mir-601 |  | 0.94081 | 1.30434 | 0.36306 | 1.108147 |
| hsa-mir-199b |  | 0.18618 | 0.22531 | 0.06098 | 0.333512 |
| hsa-mir-126 |  | 0.78463 | 1.00444 | 0.14739 | 0.460789 |
| hsa-mir-602 |  | 1.01543 | 1.40500 | 0.19507 | 0.820987 |
| hsa-mir-511-1 |  | 0.97810 | 0.88316 | 0.20580 | 1.122458 |
| hsa-mir-604 |  | 0.71105 | 1.07282 | 0.11769 | 0.902181 |
| hsa-mir-605 |  | 2.27910 | 1.03689 | 0.19339 | 0.941671 |
| hsa-mir-606 |  | 0.86918 | 1.24083 | 0.26704 | 0.908755 |
| hsa-mir-346 |  | 0.86664 | 1.11483 | 0.19197 | 0.870672 |
| hsa-mir-107 |  | 0.87695 | 1.07955 | 0.18528 | 0.848954 |
| hsa-mir-608 |  | 1.17259 | 1.12006 | 0.18712 | 0.844591 |
| hsa-mir-146b |  | 1.12248 | 1.15881 | 0.18114 | 0.787627 |
| hsa-mir-202 |  | 0.64275 | 2.44709 | 0.27982 | 1.057142 |
| hsa-mir-210 |  | 0.77586 | 1.29837 | 0.15214 | 1.328097 |
| hsa-mir-483 |  | 0.68373 | 1.68624 | 0.22785 | 0.948213 |
| hsa-mir-130a |  | 1.06262 | 2.18702 | 0.29440 | 0.926624 |
| hsa-mir-611 |  | 0.93311 | 1.48088 | 0.24618 | 0.837902 |
| hsa-mir-612 |  | 1.16890 | 1.53741 | 0.28623 | 0.891404 |
| hsa-mir-139 |  | 0.41880 | 1.68602 | 0.17439 | 0.887123 |
| hsa-mir-326 |  | 0.55230 | 1.93276 | 0.16363 | 0.95338 |
| hsa-mir-613 |  | 0.68921 | 1.06496 | 0.23686 | 0.985221 |
| hsa-mir-614 |  | 0.70222 | 1.34205 | 0.27364 | 0.944165 |
| hsa-mir-615 |  | 0.86819 | 1.31826 | 0.28906 | 0.779138 |
| hsa-mir-148b |  | 0.64558 | 1.50795 | 0.27095 | 0.690217 |
| hsa-mir-616 |  | 1.20280 | 1.15889 | 0.36031 | 0.987144 |
| hsa-let-7i |  | 0.49779 | 1.87203 | 0.75701 | 1.041928 |
| hsa-mir-617 |  | 0.96462 | 1.61105 | 0.25566 | 0.831664 |
| hsa-mir-618 |  | 3.43308 | 1.15914 | 0.38631 | 1.017452 |
| hsa-mir-492 |  | 0.87745 | 1.59622 | 0.27092 | 0.963837 |
| hsa-mir-331 |  | 0.58962 | 1.40831 | 0.24761 | 0.919463 |
| hsa-mir-619 |  | 0.82119 | 1.49429 | 0.34293 | 0.856113 |
| hsa-mir-621 |  | 1.29250 | 1.40333 | 0.38858 | 0.93447 |
| hsa-mir-622 |  | 0.73356 | 1.93538 | 0.30892 | 0.968453 |
| hsa-mir-92-1 |  | 0.82489 | 1.98777 | 0.31682 | 1.03109 |
| hsa-mir-623 |  | 1.01514 | 1.91631 | 0.39790 | 0.971326 |
| hsa-mir-624 |  | 0.69509 | 2.37920 | 0.48478 | 1.066826 |
| hsa-mir-342 |  | 0.92887 | 2.17258 | 0.42825 | 1.008069 |
| hsa-mir-345 |  | 0.63729 | 2.15880 | 0.49816 | 1.291522 |
| hsa-mir-368 |  | 0.68919 | 1.48724 | 0.33029 | 0.596788 |
| hsa-mir-203 |  | 0.43475 | 1.74363 | 0.32500 | 0.407389 |
| hsa-mir-211 |  | 1.59379 | 0.86710 | 0.43171 | 0.436645 |
| hsa-mir-626 |  | 0.85156 | 2.01131 | 0.43512 | 0.390542 |
| hsa-mir-628 |  | 0.68904 | 1.48619 | 0.46162 | 0.531053 |
| hsa-mir-190 |  | 1.14451 | 1.63696 | 0.39118 | 0.339346 |
| hsa-mir-422a |  | 0.75873 | 1.37605 | 0.37495 | 0.38415 |
| hsa-mir-630 |  | 0.48873 | 1.38645 | 0.33877 | 0.427605 |
| hsa-mir-631 |  | 1.07873 | 1.57722 | 0.46513 | 0.476959 |
| hsa-mir-184 |  | 1.88946 | 1.12095 | 0.49632 | 0.34287 |
| hsa-mir-662 |  | 0.94731 | 1.41433 | 0.41919 | 0.472271 |
| hsa-mir-328 |  | 0.54939 | 1.38449 | 0.27565 | 0.421159 |
| hsa-mir-140 |  | 0.53569 | 1.87529 | 0.40865 | 0.872812 |
| hsa-mir-22 |  | 0.60992 | 2.28840 | 0.40652 | 0.520076 |
| hsa-mir-324 |  | 0.76544 | 1.43627 | 0.33263 | 0.382486 |
| hsa-mir-33b |  | 1.08223 | 1.87855 | 0.55970 | 0.710166 |
| hsa-mir-423 |  | 0.90852 | 1.36710 | 0.32835 | 0.551754 |
| hsa-mir-632 |  | 1.02946 | 1.22303 | 0.48329 | 0.424241 |
| hsa-mir-152 |  | 1.00429 | 1.56772 | 0.49722 | 0.374734 |
| hsa-mir-142 |  | 1.08753 | 1.42018 | 0.51177 | 1.045029 |
| hsa-mir-21 |  | 1.04382 | 0.77135 | 0.30441 | 0.530586 |
| hsa-mir-633 |  | 3.58226 | 0.95270 | 0.59239 | 0.506097 |
| hsa-mir-634 |  | 1.29115 | 1.11311 | 0.35229 | 0.689961 |
| hsa-mir-635 |  | 2.55920 | 1.30265 | 0.37477 | 0.523319 |
| hsa-mir-187 |  | 1.52683 | 0.86229 | 0.38717 | 0.626306 |
| hsa-mir-122a |  | 1.93382 | 0.85624 | 0.37053 | 0.521627 |
| hsa-mir-637 |  | 1.03946 | 1.46610 | 0.29020 | 0.765991 |
| hsa-mir-638 |  | 2.61084 | 1.10547 | 0.40323 | 0.474922 |
| hsa-mir-640 |  | 0.94606 | 1.39800 | 0.28911 | 0.657621 |
| hsa-mir-330 |  | 2.36630 | 1.16218 | 0.35422 | 0.68261 |
| hsa-mir-150 |  | 1.38824 | 1.76762 | 0.31785 | 0.602963 |
| hsa-mir-643 |  | 1.41184 | 1.23667 | 0.38247 | 0.525371 |
| hsa-mir-516-1 |  | 0.88766 | 3.06058 | 0.55205 | 0.325054 |
| hsa-mir-644 |  | 1.10103 | 2.10313 | 0.46930 | 0.43164 |
| hsa-mir-499 |  | 0.57252 | 1.69751 | 0.48930 | 0.43539 |
| hsa-mir-645 |  | 0.60567 | 2.15066 | 0.39766 | 0.49315 |
| hsa-mir-646 |  | 1.82034 | 1.53869 | 0.47909 | 1.025206 |
| hsa-mir-647 |  | 0.57342 | 2.44185 | 0.34608 | 0.670515 |
| hsa-mir-155 |  | 0.84517 | 1.16048 | 0.43441 | 0.590578 |
| hsa-mir-648 |  | 0.53647 | 3.25578 | 0.67539 | 0.623388 |
| hsa-mir-185 |  | 0.53265 | 1.23272 | 0.31698 | 0.627212 |
| hsa-mir-649 |  | 1.93020 | 1.40471 | 0.40014 | 1.034886 |
| hsa-mir-651 |  | 0.91448 | 1.28811 | 0.37172 | 0.483723 |
| hsa-mir-223 |  | 0.59934 | 1.64711 | 0.40934 | 0.455065 |
| hsa-mir-384 |  | 0.37515 | 1.80145 | 0.22606 | 0.651711 |
| hsa-mir-325 |  | 0.66295 | 1.58204 | 0.27643 | 0.506732 |
| hsa-mir-361 |  | 1.43949 | 2.47220 | 0.99003 | 0.586956 |
| hsa-mir-448 |  | 0.51048 | 1.25619 | 0.34066 | 0.580201 |
| hsa-mir-450-1 |  | 0.56116 | 1.66823 | 0.27841 | 0.527392 |
| hsa-mir-504 |  | 0.49883 | 1.28964 | 0.22522 | 0.507607 |
| hsa-mir-505 |  | 0.48035 | 1.74431 | 0.23752 | 0.618353 |
